# Toward automated classification of pathological transcranial Doppler waveform morphology via spectral clustering

**DOI:** 10.1371/journal.pone.0228642

**Published:** 2020-02-06

**Authors:** Samuel G. Thorpe, Corey M. Thibeault, Nicolas Canac, Kian Jalaleddini, Amber Dorn, Seth J. Wilk, Thomas Devlin, Fabien Scalzo, Robert B. Hamilton

**Affiliations:** 1 Department of Research, Neural Analytics, Inc., Los Angeles, California, United States of America; 2 Department of Neurology, Erlanger Medical Center, Chattanooga, Tennessee, United States of America; 3 Department of Neurology, University of California Los Angeles, Los Angeles, California, United States of America; University of Warwick, UNITED KINGDOM

## Abstract

Cerebral Blood Flow Velocity waveforms acquired via Transcranial Doppler (TCD) can provide evidence for cerebrovascular occlusion and stenosis. Thrombolysis in Brain Ischemia (TIBI) flow grades are widely used for this purpose, but require subjective assessment by expert evaluators to be reliable. In this work we seek to determine whether TCD morphology can be objectively assessed using an unsupervised machine learning approach to waveform categorization. TCD beat waveforms were recorded at multiple depths from the Middle Cerebral Arteries of 106 subjects; 33 with Large Vessel Occlusion (LVO). From each waveform, three morphological features were extracted, quantifying onset of maximal velocity, systolic canopy length, and the number/prominence of peaks/troughs. Spectral clustering identified groups implicit in the resultant three-dimensional feature space, with gap statistic criteria establishing the optimal cluster number. We found that gap statistic disparity was maximized at four clusters, referred to as flow types I, II, III, and IV. Types I and II were primarily composed of control subject waveforms, whereas types III and IV derived mainly from LVO patients. Cluster morphologies for types I and IV aligned clearly with Normal and Blunted TIBI flows, respectively. Types II and III represented commonly observed flow-types not delineated by TIBI, which nonetheless deviate from normal and blunted flows. We conclude that important morphological variability exists beyond that currently quantified by TIBI in populations experiencing or at-risk for acute ischemic stroke, and posit that the observed flow-types provide the foundation for objective methods of real-time automated flow type classification.

## 2. Introduction

Transcranial Doppler ultrasound (TCD) is a noninvasive methodology for measuring Cerebral Blood Flow Velocity (CBFV) through the large arteries of the brain [1–4]. The pulsatile CBFV waveform can provide information concerning numerous cerebrovascular pathologies [5–8], including stroke [9–13], intracranial hypertension [14–17], sickle cell disease [18–20], and mild Traumatic Brain Injury [21–23]. In the context of acute ischemic stroke, the leading cause of long-term disability in the United States [24], TCD is commonly used to detect occluded and stenosed cerebral arteries [25–30]. When blood flow in these arteries is occluded, impaired oxygen supply can cause rapid brain tissue death, and permanent neurological dysfunction [31–33]. In particular, Large Vessel Occlusions (LVO) involving partial or total blockage of the Middle Cerebral and/or Internal Carotid Arteries (MCA/ICA) have disproportionately high morbidity and mortality due to the large volume of brain tissue which these vessels supply [34–36]. Because TCD provides specific information about the status of flow through the cerebral vasculature, TCD examinations are routinely conducted as standard of care at many comprehensive stroke centers [37,38].

Numerous methodologies for evaluating stroke with TCD have been published. Occlusion can often be determined by straightforward comparison of mean blood flow velocities across multiple vessels in both cerebral hemispheres [12,39–41]. However, as discussed further in subsequent paragraphs, it can also be determined by evaluating specific aspects of waveform morphology [25–30]. The morphology of the TCD signal is that of a pulsatile waveform with dominant period dictated by heart rate. Typically, each cycle consists of an initial systolic upstroke, followed by three consecutive peaks (referred to as P1, P2, and P3, respectively) which correspond to specific events in the cardiac cycle, and subsequent return to diastolic minimum [42–44]. Absence or distortion of these features may indicate flow irregularities associated with various cerebral hemodynamic pathologies such as LVO. Indeed, a growing number of studies have demonstrated changes in the timing and amplitude of waveform peaks associated with medical conditions ranging from intracranial hypertension [43,45,46] to various age-related changes and pharmacological interventions [47,48].

The most widely-cited method for assessing stroke with TCD is the evaluation of waveform morphology using Thrombolysis in Brain Ischemia (TIBI) flow grades [25,27]. In this framework, waveform features such as the presence of identifiable pulses/peaks and the onset of maximum velocity are used to determine grade assignment [26,28–30]. The TIBI categories range from grades 0–5, with 5 indicating normal flow, and grades 0 and 1 designating absent (0) or minimal (1) flow associated with complete or partial vascular occlusion. The grades between are associated with characteristic morphologies which are used to determine blunting (grade 2), dampening (grade 3), or stenotic (grade 4) flows. Several foundational studies have shown TIBI assessment to be a valuable tool for LVO diagnosis, possessing sensitivity and specificity often exceeding 90% [5,25,30] relative to gold standard Computed Tomography Angiography (CTA). However, reliable determination of TIBI grades requires subjective assessment by expert evaluators, severely limiting their utility for prehospital stroke assessment by less specialized personnel.

The complexity and subjectivity inherent in the assignment of TIBI categories, along with their demonstrable utility for stroke assessment, make them a natural candidate problem for automation via machine learning. That is, the clinical relevance of the morphological information captured by subjective TIBI grading heuristics has now been clearly established, but an objective and computationally tractable framework for extracting this information does not currently exist. Though the traditional TCD Pulsatility Index constitutes an objective metric which describes waveform morphology to some degree, the information it contains is too coarse to effectively detect the presence of occluded or stenosed vessels [28,49]. Moreover, machine learning approaches to extracting information from TCD waveforms have already proven fruitful for multiple clinical applications [50–56], including the diagnosis of cerebrovascular stenosis [57]. In particular, recent work by our group has shown that a TCD-derived morphological biomarker termed Velocity Curvature Index (VCI) may provide a robust, objectively computable metric for detecting Large Vessel Occlusion (LVO) [54,58]. Though VCI readily identifies waveforms with pathologically deviant curvature, it does not differentiate between pathological morphologies such as those delineated by the TIBI scale. An objective means of waveform categorization could thus provide additional information concerning stroke etiology to better inform stroke triage and transfer decisions.

Before approaching waveform categorization explicitly as a classification problem, a more foundational issue must first be addressed, in that it is currently unclear to what degree the TIBI categories actually capture the natural variance inherent in empirical data. That is, we know these categories are useful, but we do not know that they are comprehensive. Additional waveforms may be present which are also informative, and perhaps a different subset may better explain variability across subjects. In this work we take a data-driven approach to waveform categorization, retrospectively applying an unsupervised learning algorithm to a dataset comprised of multiple subject groups, including patients experiencing acute LVO, as well as control subjects collected both in and out of hospital. For these purposes we employ spectral clustering, which does not make strong assumptions about inherent cluster density, and thus performs well when clusters are connected but potentially non-convex [59–61]. Combined with gap statistic criterion for choosing the optimal cluster number [62], this approach yields a natural partitioning of waveforms into groups with distinct morphological characteristics.

For clustering, we sought to include information of clinical relevance to the presence of LVO, thus we chose morphological features which quantify important aspects of TIBI evaluation criteria (see section 3.3 for details). The *onset* feature encodes information concerning where in the cardiac cycle the maximal velocity is attained, which can be important in distinguishing blunted waveforms (TIBI grade 2) from healthy (TIBI grade 5) [25]. This feature is a time-normalized version of the “latency to P1” variable used in previous studies of TCD morphology [45,46]. Likewise, the *canopy* feature provides information about the size of the systolic complex, which is helpful for distinguishing both blunted and stenotic waveforms from healthy. This feature is similar to the “fractional time in systole” used to evaluate waveform morphology in [43]. Finally, the number and prominence of observed peaks/troughs (quantified by our *peaks* feature) are also critical in distinguishing blunted and stenotic waveforms from healthy. We hypothesize that LVO subject waveforms will fall into clusters which are mostly distinct from non-LVO controls, for which we can subsequently compare alignment with established TIBI categories.

## 3. Materials and methods

### 3.1. Subjects

We compared TCD waveform morphology across three subject groups: one with CTA-confirmed LVO, a second non-LVO control group collected in-hospital, and a third group of control subjects collected out of hospital. LVO and in-hospital controls (IHC) were enrolled at Erlanger Southeast Regional Stroke Center in Chattanooga, TN, between October 2016 and October 2017. As detailed in [54,58], subjects who arrived at the hospital presenting with stroke symptoms received TCD examinations along with standard care, pharmaceuticals and CT imaging. CTA was performed using a GE Lightspeed VCT 64-section multidetector scanner (GE Healthcare, Milwaukee, WI) with a slice thickness of 0.625 mm, and bolus injection of 70–150 mL of Omnipaque 350 (GE Healthcare, Milwaukee, WI) contrast material (4.0 mL/s). CTA images were reformatted in the coronal and sagittal plane, and 10-mm maximum intensity projection reconstructions were rendered for review. Occlusion location was determined by the radiologist on call, and reviewed/confirmed independently by the authors. TCD examinations were performed during available time between patient testing/treatment, and in no way impacted patient care. Subjects for whom an acceptable exam was obtained within 4 hours of imaging, and to whom no Table 1 (left column) exclusion criteria applied, were eligible for enrollment in either the LVO group (if CTA confirmed occlusion of the proximal extracranial or terminal intracranial ICA segments, or M1/M2 branches of the MCA), or the IHC group (if no LVO were detected). Out of hospital controls (OHC) were scanned at multiple locations in Los Angeles, CA, and did not undergo CTA examination. Volunteers were eligible for enrollment if between 40–85 years of age, and all Table 1 (right column) exclusion criteria were absent. Experiment protocols for LVO and IHC subjects were approved by the University of Tennessee College of Medicine Institutional Review Board (ID: 16–097), and for OHC subjects by Western Institutional Review Board (ID: 20151682). The study was carried out in accordance with the recommendations of the Declaration of Helsinki, with written informed consent from all subjects.

**Table 1:** Subject exclusion criteria.

Exclusion Criteria	
LVO, IHC subjects	OHC subjects
**1.** Head CT findings consistent with acute primary intracranial hemorrhage (SAH, ICH, etc.).	**1.** Individuals taking any psychoactive medication.
**2.** Hemodynamically unstable patients requiring pharmacological support for hypotension.	**2.** Systolic arterial blood pressure greater than 140 mmHg (Hypertensive).
**3.** Subjects who underwent partial or full craniotomy.	**3.** Individuals presenting with an external wound in head.
**4.** Additional intracranial pathologies present (tumor, hydrocephalus, etc.).	**4.** Pregnant women.
**5.** Anticipated insufficient time to acquire a complete set of scan as described by the protocol.	**5.** Individuals who have a known history of: severe TBI, moderate TBI (within previous 3 years), vascular disease, stroke, sickle cell anemia, brain tumor, epidural or subdural hematoma, abnormal MRI or CT scan of the brain, cardiovascular disease.
**6.** Significant hemodynamic pharmacological agent (cocaine, amphetamine, etc.).	
**7.** Subjects who are under arrest for a felony.	

Subject exclusion criteria. Note the following abbreviations: SAH–Subarachnoid Hemorrhage, ICH–Intracranial Hemorrhage, TBI–Traumatic Brain Injury, MRI–Magnetic Resonance Imaging, CT–Computerized Tomography.

### 3.2. TCD waveform recording & processing

CBFV signals were acquired by trained technicians using 2 MHz handheld probes in conjunction with either DWL Doppler Box-X (DWL Inc., USA), or Lucid M1 TCD System (Neural Analytics Inc., USA), to transtemporally insonate the left/right MCA. The technician was instructed to obtain recordings for as many depths as possible between 45–60 mm in both the left/right cerebral hemispheres, with the depth range enforced to help ensure errant recordings of other vessels (e.g. ACA/PCA or M2) were not included in the analysis. Once signal was identified and optimized at a specific depth, waveform recordings were made in 30-second intervals. The choice of 30 seconds was intended to guarantee the acquisition of a sufficiently large ensemble of at least 15 individual beat waveforms from which to compute an average beat waveform for analysis. The choice of 15 individual beats is a free parameter chosen to optimize the trade off between the size of the acquired ensemble and the technician’s available time and ability to maintain continuous steady contact between the probe and the subject’s head.

To construct the average waveform for each recorded depth, individual beat waveforms from each recording were extracted offline (post-recording) using an automated beat identification algorithm [63] to identify the samples marking the onset of each beat (and thus the end of the previous beat). Outlier beats with excessive artifact and/or poor signal quality were automatically identified and rejected via Iterated Interquartile Range exclusion with cross-correlation and beat length as primary comparators [64]. To be included in this analysis, exams were required to contain at least one bilateral pair of left/right MCA scans at depths between 45–60 mm, each containing at least 15 accepted beats. To obtain a rectangular ensemble array for averaging, the accepted beats were aligned at the onset of systolic upstroke and individually padded with each beat’s final value to the length of the longest beat. The resulting ensemble array was averaged across beats, resulting in a single representative average beat waveform for each recorded 30 second interval. It is very important to note that waveforms were only averaged over beats obtained from individual depth recordings (i.e. consecutive beats recorded in a single contiguous 30 second interval), and were not averaged across different recordings or across the left/right hemispheres. OHC waveforms, digitally sampled at 400 Hz, were resampled to 125 Hz to match the native sampling rate of LVO and IHC waveforms. To reduce the potential effects of artifactual noise in the individual beats of higher frequency than the relevant hemodynamics (roughly 10 Hz), all waveforms were smoothed via convolution with a 90 ms Hanning window. Since this analysis sought to evaluate morphological commonality regardless of underlying heart rate or velocity scale, each waveform was normalized with respect to both time and velocity. These normalizations help ensure that waveforms with generally similar shapes, but potentially different heart rates or velocity ranges will still likely be clustered together. The velocity normalization was accomplished for each waveform by first subtracting the minimum, then subsequently dividing by the resultant maximum, thus rescaling to the interval [0, 1] along the velocity axis. The temporal normalization was accomplished by resampling each waveform to 100 total samples (via cubic spline), effectively enforcing a common heart rate across waveforms.

### 3.3. Cluster feature extraction

For clustering, we sought to construct a feature space of minimal dimension which would nonetheless capture enough stroke related morphological variance to produce a meaningful clustering partition for comparison to TIBI. Clearly, there are a vast number of morphological variables which might be used for this purpose. For example, the Morphological Clustering Analysis of Intracranial Pressure Pulses (MOCAIP) algorithm [65] defines 128 morphological features which can be extracted from TCD and pressure wave pulses [45]. However, most of these assume that the standard peaks/troughs associated with healthy waveforms are present and identifiable. For pathological waveforms such as those associated with LVO, this assumption is rarely met. As noted in Section 1, our strategy was to start with the features we deemed most relevant to flow grade assignment using the TIBI criterion. These features must also be readily computable for all pulsatile TCD waveforms, regardless of the presence of stroke pathology. The three features decided upon for this analysis meet these criteria, but admittedly represent a first approach to the problem which could be expanded or refined in future work.

**Fig 1:**
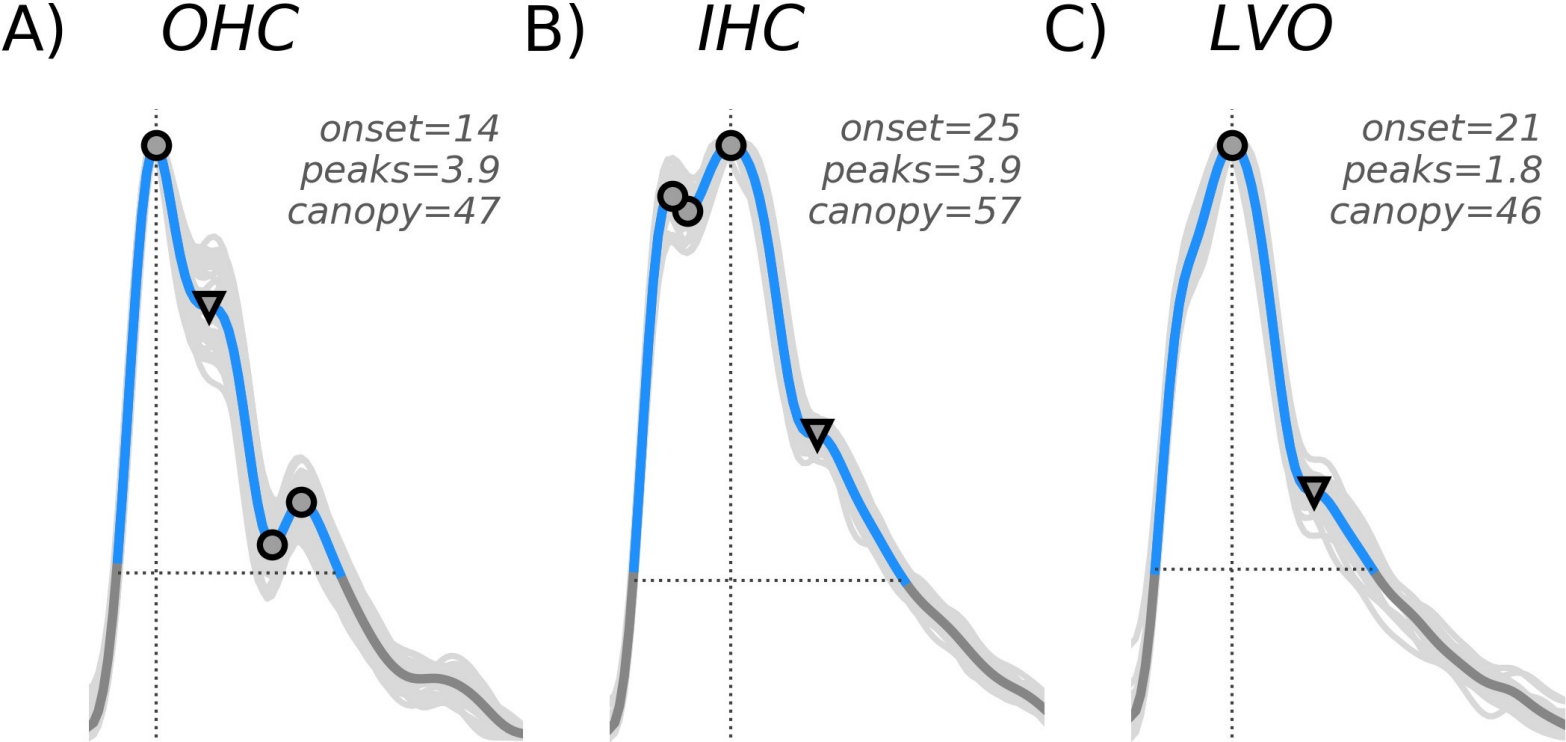
Cluster features. Cluster features are depicted for three example waveforms taken from each of the subject groups; OHC (A), IHC (B), and LVO **(C)**. All waveforms were normalized in both time and velocity, so as to span 100 total samples ranging from zero to one on the y-axis. The onset variable (vertical line) marks the time sample where maximum velocity is attained. The canopy variable (horizontal line) marks the length (in samples) of the systolic canopy. The *peaks* variable is a weighted sum of waveform peaks, both true (indicated by circles) and pseudo (triangles).

From each waveform, denoted *x(t)* in Eqs 1–3, we extracted each of the three morphological features (depicted in Fig 1). The first, termed *onset* (Eq 1), marked the temporal onset of maximal velocity. The second feature, termed c*anopy* (Eq 2), was defined as the number of samples comprising the systolic complex, i.e. the “beat canopy”, given by the cardinality of the set of samples with velocity greater than 25% of the diastolic-systolic range (see [58] for details). The final feature, termed *peaks* (Eq 3), quantified the number and “weight” of waveform peaks/troughs. First we identified the set of true-peaks/troughs (TP, approximate zeros of the first derivative) as points in the canopy corresponding to a sign-change in the difference between successive samples. True peaks were each assigned a weight of one. Next we identified the set of “pseudo-peaks/troughs” (PP, points where the derivative is small but non-vanishing) where the difference magnitude between successive samples dropped below a chosen critical threshold of 0.01 (choosing the point with smallest difference magnitude in any group of adjacent sub-threshold samples). For pseudo-peaks we assigned weights corresponding to one minus the ratio of the associated difference magnitude to the threshold (0.01), such that those with the smallest derivative were weighted most heavily. The *peaks* feature was then computed as the sum over all corresponding weights. The *onset*, *canopy*, and *peaks* features for each waveform are defined explicitly by Eqs 1–3, respectively. Note that the “card” expression in Eqs 2 and 3 refers to the cardinality of a set, and the colons in Eqs 1 and 2 denote the set- theoretic notion of “having the property such that”. In Eq 2, *t*_*systolic*_ and *t*_*diastolic*_ refer to the time samples at which the waveform attains its systolic maximum and diastolic minimum, respectively.

$$onset=n:x(t_{n})=\underset{i\in \{1,2,...100\}}{max}\{x(t_{i})\}$$ (1)

$$canopy=card(\left\{i:x(t_{i})>x(t_{0})+0.25\left(x(t_{systolic})-x(t_{diastolic}\right))\}\right)$$ (2)

$$peaks=card(TP)+\sum_{k\in PP}1-\frac{\left|x(t_{k})-x(t_{k-1})\right|}{0.01}$$ (3)

With regard to specification of the threshold 0.01 in Eq 3, the idea is to allow for adequate consideration of “pseudo peaks/troughs” for which the derivative gets very small, but doesn’t quite vanish, and weight them towards the total *peaks* count. Our experience with the data has shown that there are indeed numerous cases for which this threshold adds meaningful variance to the peaks feature distribution. For example, each waveform depicted in Fig 1 possesses a pseudo-peak, occurring at roughly the location expected for either the P2 (Fig 1A), or P3 (Fig 1B and 1C); we refer the reader to the introduction for background on the peak structures (P1, P2, and P3) typically observed sequentially in standard TCD beat morphology. If these were idealized waveforms with true peaks and corresponding true troughs at these locations, the combined structures would count two units toward the *peaks* value. Without consideration of pseudo-peaks, these structures would contribute zero units towards the total, which we believe to be less descriptive and ultimately less meaningful, whereas the specification we have made allows them an intermediate contribution of 0.9, 0.9, and 0.8 for 1A, B, C, respectively. The smaller this threshold is chosen, the closer the peaks feature comes to approximating the discrete count of the true peaks, *TP*, for which the derivative does vanish.

### 3.4. Spectral clustering and gap analysis

In order to standardize the ranges of the resultant feature distributions for clustering, z-score normalization (not to be confused with the z-transform used in signal processing) was applied across observations [66,67]. That is, the feature distributions were rescaled by subtracting from each feature value the mean taken across all observations, and subsequently dividing by the standard deviation across observations. The resultant three-dimensional feature space was then partitioned via spectral clustering implemented in scikit-learn, an open-source machine learning library for Python [68], specifically the *SpectralClustering* module with default parameters and radial basis kernel. To determine the optimal number of clusters, we employed the gap statistic methodology of Tibshirani et al. [62], which is widely used for cluster number selection [69,70]. Conceptually, the gap statistic was designed to formalize the statistical notion of finding the “elbow” in the graph of cluster dissimilarity as a function of cluster number within a widely applicable nonparametric framework; the critical underlying idea being that consideration of additional clusters beyond the optimal number should yield diminishing returns in terms of minimizing cluster dispersion (i.e. the sum across clusters of pair-wise distances between members of each cluster). For our analysis, we compared gap statistics (*G*_*k*_) for total clusters (*k*) ranging from two to seven. In this procedure, gap statistics are computed as the difference between observed log intra-cluster dispersion pooled across *k* clusters (denoted *W*_*k*_), and the analogous expected dispersion bootstrapped from a null distribution incorporating the covariance structure of the observed data. As detailed in method b (section 4) of [62], each of the 1000 bootstrap iterations was generated by sampling uniformly over the range of the columns of the observed data transformed by its right-singular vectors, and back-transforming the resultant sample to feature space via the right-singular transpose. The optimal number of clusters were selected as the smallest *k* such that *G*_*k*_ > *G*_*k+1*_ –*S*_*k+1*_, where *S*_*k*_ is the standard deviation of the *k*-cluster bootstrap distribution corrected to account for simulation error.

### 3.5. Cluster archetypes

To visualize the characteristic morphology of the resultant clusters, we first computed for each cluster the matrix of squared Euclidean distances between all cluster member waveforms (i.e. the entities denoted as *d*_*ii*_ in [62]), and ranked each by average distance to other members. For each cluster, the individual waveform with the smallest mean intra-cluster distance was designated as the most representative exemplar. To further visualize commonalities in cluster morphology independent of an individual exemplar, the five waveforms with smallest mean intra-cluster distance were averaged together to obtain the waveform archetype for each cluster.

## 4. Results

### 4.1. Subject demographics

The current analyses included 33 LVO subjects (16 female), 33 IHC subjects (13 female), and 40 OHC subjects (25 female), with average ages of 66.9 (SD = 15.7), 56.4 (SD = 16.3), and 58.4 (SD = 10.9) years, respectively. A total of 996 average beat waveforms were included in this analysis, with 354, 445, and 196 contributed by LVO, IHC, and OHC subjects, respectively, resulting in a clustering feature space of dimension 996 x 3.

### 4.2. Gap analysis & cluster archetypes

**Fig 2:**
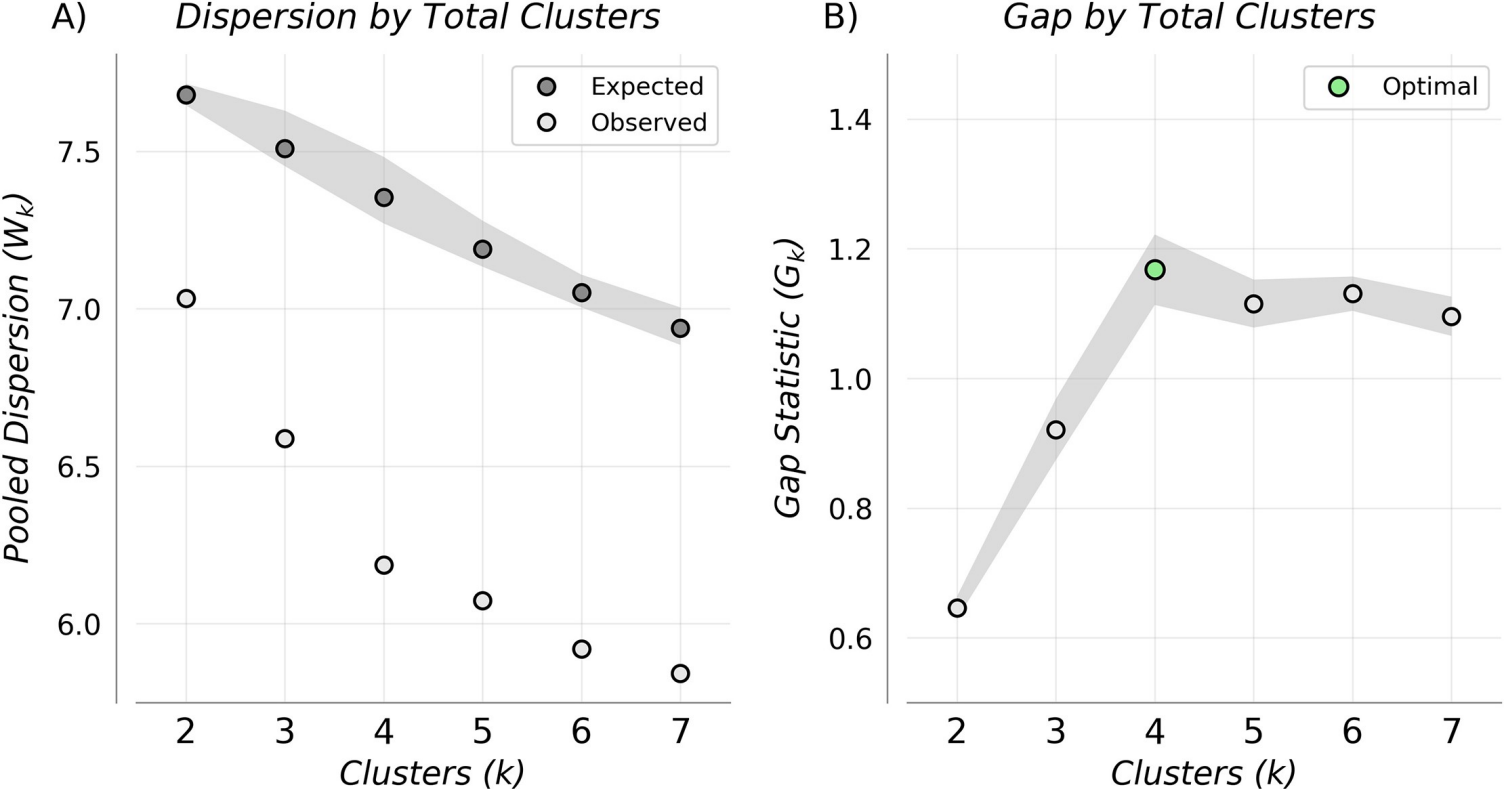
Cluster dispersion and gap statistic. Pooled Intra-cluster dispersion (A), and associated Gap Statistics (B) as a function of cluster number. Gap-statistic disparity was maximized at four clusters which also corresponded to the optimal number given by the selection criteria in Tibshirani et al. [19].

**Fig 3:**
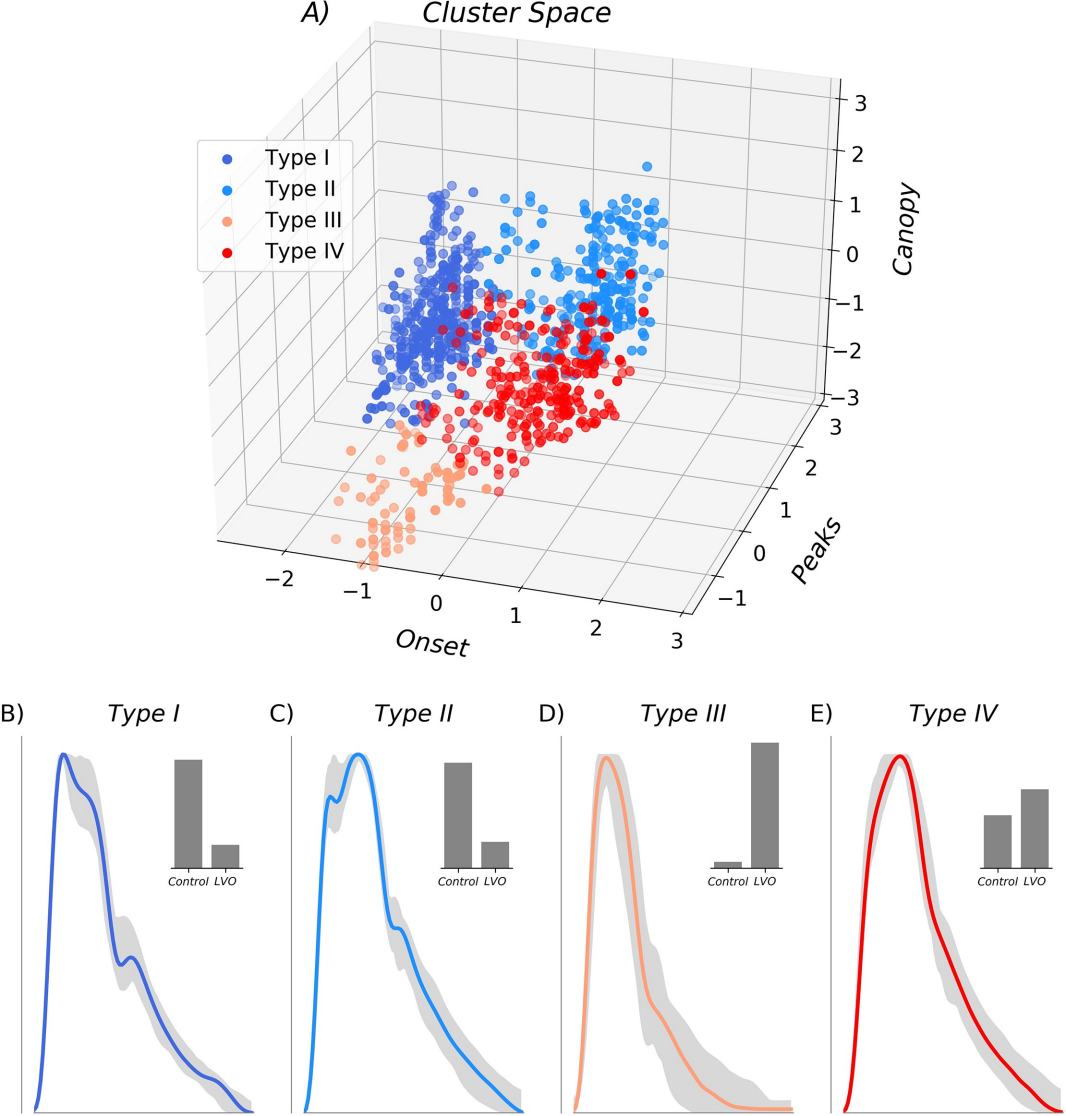
Cluster feature space. Three-dimensional feature space is shown in Z-scored coordinates with the optimal four clusters indicated by color. Associated cluster morphologies are shown for each beat type (B-E), with each cluster archetype shown in color, and the range of the next 50 most representative exemplars depicted in gray. Associated histograms in the upper right (B-E) demonstrate that Type I and II clusters were primarily composed of waveforms from control patient populations, whereas Type III and IV clusters were primarily composed of LVO patient waveforms.

Fig 2A shows expected and observed log intra-cluster dispersion as a function of total clusters, with the gap statistic determined by their difference. The elbow in observed dispersion at four clusters corresponds to both the maximum gap statistic (Fig 2B) and optimal number of clusters determined by the Tibshirani et al. criteria [19]. The associated feature space (Fig 3A) is shown partitioned into the four resultant clusters, with membership indicated by color, and associated cluster archetypes shown in Fig 3B–3E. The largest cluster (type I), containing 400 waveforms, was characterized by early max velocity onset with wide canopy and strong peaks (Fig 3B). Of waveforms in this cluster, 18% came from LVO subjects, with 82% from controls (52% IHC, 30% OHC). The second largest cluster (type II), containing 257 waveforms, was characterized by later max velocity onset, with wide canopy and strong peaks (Fig 3C). Of waveforms in this cluster, 20% came from LVO subjects versus 80% from controls (68% IHC, 12% OHC). The smallest cluster (type III), containing 83 waveforms, was characterized by early max velocity onset, with narrow canopy and weak peaks (Fig 3D). Of waveforms in this cluster, 95% came from LVO subjects versus 5% from controls (1% IHC, 4% OHC). The final cluster (type IV), containing 256 waveforms, was characterized by late max velocity onset, with wide canopy but weak peaks (Fig 3E). Of waveforms in this cluster, 60% came from LVO subjects versus 40% from controls (28% IHC, 12% OHC).

## 5. Discussion

### 5.1. Comparison to TIBI flow grades

**Fig 4:**
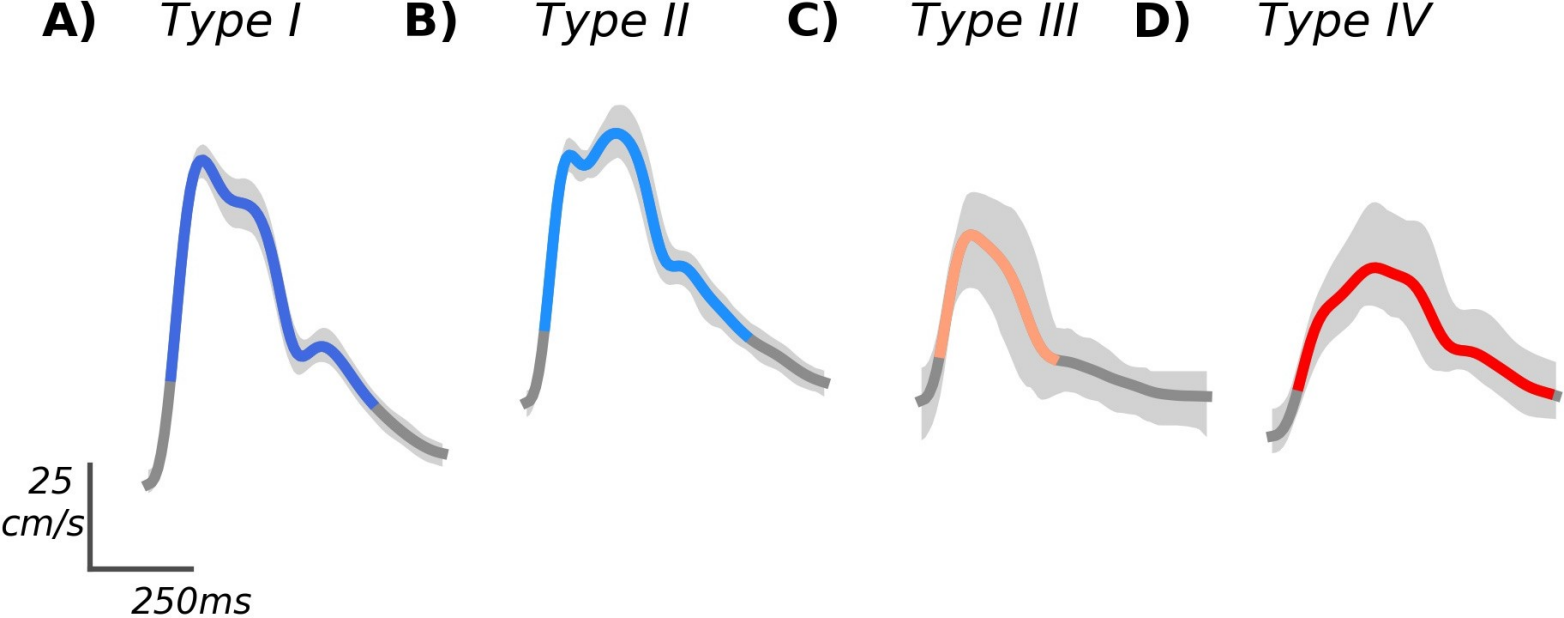
Example waveforms. The individual example waveforms most representative of each cluster are shown in standard units (unnormalized), with variability across individual beats depicted in light gray. The Types I and II examples (A and B) originated from the OHC and IHC groups, respectively, whereas the Types III and IV examples (C and D) originated from LVO, and IHC subjects, respectively.

Of the clusters we observed, two have definitive analogues on the TIBI scale. Specifically, our type I cluster showed the early systolic maximum and recognizable peak structure associated with TIBI grade 5 normal flow. Similarly, our type IV cluster exhibited the delayed flow acceleration with no discernible early peak, and maximum velocity in mid-to-late systole, characteristic of TIBI grade 2 blunted flow; features which can also be clearly observed in the most representative cluster waveforms, shown unnormalized in Fig 4. Accordingly, we observed type I flows more often in control subjects, whereas type IV flows were more commonly associated with LVO. The remaining clusters did not have unambiguous TIBI analogues, though their contrasting subject group compositions suggest type II is more commonly observed in controls, and type III nearly always associated with LVO. The type II cluster, characterized by late onset maximal velocity but otherwise normal peaks, may reflect differences in peripheral vascular resistance relative to type I, which could conceivably impact either or both the initial systolic upstroke and/or the timing of reflected waves affecting the amplitude of the mid-systolic peak.

Interpretation of the pathological type III morphology represents the most speculative aspect of this work. We infer from its strong association with LVO subjects that it likely results from occlusion or stenosis of the cerebral vessels, though in a manner distinct from typically blunted waveforms, leaving the initial systolic acceleration unaffected while suppressing all subsequent morphological structure. These features can be seen clearly in both the associated cluster archetype (Fig 3D), as well as the most representative cluster exemplar (Fig 4C). This pattern is intriguingly similar to previously observed waveforms associated with vascular stenosis (see, for example, Fig 4 in [71]), suggesting this flow type may rightly be thought of as the analog of the TIBI stenotic grade 4. However, multiple reasons compel us to be cautious with this claim. First, this flow type was by far the smallest cluster, which was manifest in only a small number of subjects. CTA case reports for most of these subjects did indicate significant, but non flow-limiting stenoses. However, such moderate stenoses are not uncommon in the LVO subject population in general. Moreover, the TIBI grade 4 categorical definition given by Demchuk et al. [27] refers both to elevated velocity relative to the adjacent (unaffected) hemisphere, as well as evidence for flow turbulence, which were not quantified as part of this analysis and thus require further work to assess. Finally, previous studies have shown that qualitatively similar TCD morphologies can be associated with intracranial hypertension and/or elevated cerebral perfusion pressure [43], which were not controlled for in this study. For the time being we reserve judgment as to whether future studies incorporating richer spectral data might confirm this flow type to have stenotic etiology.

### 5.2. Limitations and future work

Considering the remaining TIBI flow grades, the lowest are not associated with sufficiently pulsatile CBFV waveforms, and thus could not be represented in our data set. Specifically, grade zero is defined as the absence of flow, whereas grade 1 (minimal flow) is so weakly pulsatile as to give rise to nearly flat waveforms when averaged over successive beats. In this study we required subjects to exhibit bilateral pulsatile data to be included in analysis. This was done to ensure that absence of temporal acoustic windows would not be mistaken for absence of MCA flow. However, future experimental protocols could be modified to require TCD examination of all cerebral vessels in the anterior circulation. Evidence of flow in any of these vessels in a given hemisphere without corresponding evidence of adjacent MCA flow would allow for confident assessment of absent or minimal flow grades, likely bringing our current results further into alignment with the TIBI scale. The remaining TIBI grade 3 flow (Dampened), is not solely morphologically defined, requiring comparison of velocity magnitude relative to a control waveform for assignment, and thus cannot clearly align with our clusters. Future work could explore whether our clustering framework might be extended for application to sets of waveforms, including relative velocities as features, which might help reconstruct these other TIBI categories.

From the 3-dimensional cluster space (Fig 3A) it is clear that adjacent cluster types II and IV share a fuzzy boundary primarily determined by the peaks variable, suggesting the two can be difficult to differentiate when systolic peaks are not clear. Indeed, the type IV cluster had the least homogeneous group composition, with 40% of waveforms originating from control subjects; a fact which would negatively impact specificity were we to use these clusters alone to classify LVO. Underlining this point is the fact that the most representative exemplar for both the type II and type IV waveforms originated from the IHC group. Clearly, further work is needed to determine whether additional or refined clustering features, perhaps derived from waveform spectrograms and associated M-mode, might help disambiguate these groups.

### 5.3. Conclusions

To our knowledge, this study presents the first unsupervised learning analysis of LVO pathology evident in the TCD signal. We have shown that spectral clustering can readily recover meaningful TCD flow types bearing clear relation to known morphological categories. Moreover, the resultant cluster archetypes provide definitive morphological templates, enabling automated categorization of novel waveforms via numerous potential comparative methods, the refinement of which should provide fruitful avenues for future work. Ultimately, we will explore whether such automated labels can be combined with other established metrics, such as VCI and velocity asymmetry, to improve LVO classification efficacy.
